# A New Class of Chiral Polyethers and Polyesters Based on the [2.2]Paracyclophane Scaffold

**DOI:** 10.3390/polym16111603

**Published:** 2024-06-05

**Authors:** Patrick Kern, Henrik Tappert, Stefan Bräse

**Affiliations:** 1 Institute of Organic Chemistry (IOC), Karlsruhe Institute of Technology (KIT), Kaiserstrasse 12, 76131 Karlsruhe, Germany; patrick.kern@kit.edu (P.K.); henrik.tappert@kit.edu (H.T.); 2 Institute of Biological and Chemical Systems-Functional Molecular Systems (IBCS-FMS), Karlsruhe Institute of Technology (KIT), Kaiserstrasse 12, 76131 Karlsruhe, Germany

**Keywords:** chiral polymer, [2.2]paracyclophane, polyester, polyether, epoxide

## Abstract

Over the past decades, the research on optically active polymers (OAPs) has significantly grown, and extensive studies have been carried out on their syntheses, conformations, and applications. The most commonly used OAPs are based on natural products such as sugars or amino acids, which limits their scope. A broader range of applications can be achieved by synthesizing lab-tailored monomers, which allow precise control over structure and properties. This research developed a four-step synthetic route to a previously unreported chiral [2.2]paracyclophane-based epoxide monomer. An aluminum catalyst and an alkylammonium initiating system were applied and optimized for its polymerization to provide access to a novel class of chiral polyethers. Furthermore, we demonstrated the copolymerization viability of the (4-[2.2]paracyclophanyl)oxirane monomer using phthalic anhydride.

## 1. Introduction

Chirality accompanies us in almost every aspect of our lives. This is evident considering the chirality of the approximately 800 drugs obtained from natural sources. Aside from 2% of racemic and 1% of achiral drugs, 97% of those drugs are chiral [1]. The reason for this is the dependency of life on optically active macromolecules, such as nucleic acids, proteins, and polysaccharides [2]. One of the simplest applications of chiral polymers is their usage as chiral stationary phases for gas and liquid chromatography. This technique is particularly important in the purification process of chiral pharmaceuticals since different enantiomers of the same compound can have different effects on the human body [3]. Most chiral polymers used for this purpose are based on enzyme-made natural products, which limits the scope of available polymers. In contrast, lab-made polymers with precise bottom-up designs allow full control over structure, properties, and functions. This is particularly important for areas where minute control over properties is key, such as functional materials for chiral optoelectronics or catalysis [4,5]. For this reason, the synthesis of artificial optically active polymers became an important scientific goal. This work aims to expand the scope of usable and easily modifiable scaffolds by introducing [2.2]paracyclophane (PCP) as a building block. This unique molecule can be utilized as a simple source of chirality since it exhibits planar chirality upon substitution. PCPs are versatile molecules with a broad area of application because of their fascinating properties, e.g., their electronic structure and inherent planar chirality [6]. As a result, they are a prevalent 3D scaffold that is covered in a great number of reviews [6,7,8,9,10]. The versatility of these intriguing PCP derivatives, ranging from catalysis for asymmetric synthesis [11] over materials [7] up to photoredox catalysis [12], was demonstrated extensively. Despite that, only a few polymers using an intact, chiral PCP core are reported, all using racemic materials [13,14]. In this work, enantiomerically pure PCP was used to synthesize the epoxide (4-[2.2]paracyclophanyl)oxirane to demonstrate the feasibility of the concept for the important class of polyethers. Furthermore, the demonstration was extended by copolymerization with phthalic acid to yield polyesters.

## 2. Materials and Methods

### 2.1. General Remarks

The starting materials, solvents, and reagents were purchased from abcr (Karlsruhe, Germany), Acros (Geel, Belgium), Bernd Kraft (Duisburg, Germany), arbosynth (Redcar, United Kingdom), ChemPUR (Karlsruhe, Germany), Honeywell (Offenbach, Germany), Merck (Darmstadt, Germany), Sigma Aldrich (St. Louis, MO, USA), TCI (Tokyo, Japan), and Thermo Fisher Scientific (Waltham, MA, USA) and used without further purification unless stated otherwise.

Solvents of technical quality were purified by distillation or with the solvent purification system MB SPS5 (acetonitrile, dichloromethane (DCM), diethyl ether, tetrahydrofuran, toluene) from MBraun (Garching, Germany). Solvents of p.a. quality were purchased from Acros (Geel, Belgium), Fisher Scientific (Waltham, MA, USA), Sigma Aldrich (St. Louis, MO, USA), Roth (Karlsruhe, Germany), or Riedel-de Haën (Seelze, Germany) and were used without further purification.

Air- and moisture-sensitive reactions were carried out under an argon atmosphere in oven-dried glassware using standard Schlenk techniques.

Liquids were added with a stainless-steel cannula, and solids were added in powdered shape.

Reactions at low temperatures were cooled using flat dewars produced by Isotherm (Karlsruhe, Germany) with water/ice or isopropanol/dry ice mixtures.

Solvents were evaporated under reduced pressure at 45 °C using a rotary evaporator. For solvent mixtures, each solvent was measured volumetrically.

Flash column chromatography was performed using Merck (Darmstadt, Germany) silica 60 (0.040 × 0.063 mm, 230–400 mesh ASTM) and quartz sand (glowed and purified with hydrochloric acid).

#### 2.1.1. Reaction Monitoring

All reactions were monitored by thin-layer chromatography (TLC) using silica-coated aluminum plates (Merck (Darmstadt, Germany), silica 60, F254). UV active compounds were detected with a UV lamp at 254 nm and 366 nm excitation.

GC-MS (gas chromatography–mass spectrometry) measurements were performed on an Agilent Technologies (Waldbronn, Germany) model 6890 N (electron impact ionization), equipped with an Agilent 19091S-433 column (5% phenyl methyl siloxane, 30 m, 0.25 μm) and a 5975B VL MSD detector with a turbopump. Helium was used as a carrier gas.

#### 2.1.2. Melting Point

Melting points were detected on an OptiMelt MPA100 device from the Stanford Research System (Sunnyvale, CA, USA).

#### 2.1.3. Optical Rotation

Optical rotation was measured with a Perkin Elmer (Rodgau, Germany) 241 Polarimeter using a 100 mm glass cell, a suitable solvent at the sodium-D-lines (589.0 and 589.6 nm), and a constant temperature of 20 °C.

#### 2.1.4. Nuclear Magnetic Resonance Spectroscopy (NMR)

NMR spectra were recorded on a Bruker (Karlsruhe, Germany) Avance 400 NMR instrument at 400 MHz for ^1^H NMR, 101 MHz for ^13^C NMR, or a Bruker Magnet System 600 MHz/54 mm NMR instrument at 600 MHz for ^1^H NMR.

The NMR spectra were recorded at room temperature in deuterated solvents acquired from Eurisotop (Saint-Aubin, France). The chemical shift *δ* is displayed in parts per million [ppm], and the references used were the ^1^H and ^13^C peaks of the solvents themselves as follows:

*d_1_*-chloroform (CDCl_3_): 7.26 ppm for ^1^H and 77.16 ppm for ^13^C

For the characterization of centrosymmetric signals, the signal’s median point was chosen for multiplets in the signal range. The following abbreviations were used to describe the proton splitting pattern: d = doublet, t = triplet, m = multiplet, dd = doublet of a doublet, ddd = doublet of a doublet of a doublet, dddd = doublet of a doublet of a doublet of a doublet, and dt = doublet of a triplet. Absolute values of the coupling constants “*J*” are given in Hertz [Hz] in absolute value and decreasing order. Signals of the ^13^C spectrum were assigned by distortionless enhancement by polarization transfer (DEPT) spectra DEPT90 and DEPT135 or phase-edited heteronuclear single quantum coherence (HSQC). They were specified in the following way: + = primary or tertiary carbon atoms (positive phase), − = secondary carbon atoms (negative phase), and C_q_ = quaternary carbon atoms (no signal).

#### 2.1.5. Infrared Spectroscopy (IR)

The infrared spectra were recorded with a Bruker Alpha P instrument. All samples were measured by attenuated total reflection (ATR). The positions of the absorption bands are given in wavenumbers *ṽ* in cm^−1^ and were measured in the range from 3600 cm^−1^ to 500 cm^−1^.

Characterization of the absorption bands was performed in dependence of the absorption strength with the following abbreviations: vs (very strong, 0–9%), s (strong, 10–39%), m (medium, 40–69%), w (weak, 70–89%), and vw (very weak, 90–100%).

#### 2.1.6. Mass Spectrometry (MS)

Electron ionization (EI) and fast atom bombardment (FAB) experiments were conducted using a Finnigan, MAT 95 (70 eV) instrument, with 3-nitrobenzyl alcohol (3-NBA) as the matrix and reference for high resolution. For the interpretation of the spectra, molecular peaks [M]^+^, peaks of protonated molecules [M + H]^+^, and characteristic fragment peaks are indicated with their mass-to-charge ratio (*m*/*z*) and their intensity in percent, relative to the base peak (100%), is given. In the case of high-resolution measurements, the maximum tolerated error is ±5 ppm.

#### 2.1.7. Gel Permeation Chromatography (GPC)

For GPC measurements, a PSS SECcurity2 GPC-System with AGILENT infinity 1260 II hardware was used. The device uses a refractive index detector and runs on THF as a polar phase with a flow rate of 1 mL/min at 30 °C. The used column system consists of a PSS SDV analytical column (3 μm, 300 × 8.0 mm^2^, 1000 Å) with a PSS SDV analytical precolumn (3 μm, 50 × 8.0 mm^2^). Poly(methyl methacrylate) with masses ranging from 102 to 62,000 Da were used for calibration.

### 2.2. Synthetic Procedures

**(rac)-4-Acetyl [2.2]paracyclophane (2)** [15]

To a solution of [2.2]paracyclophane (**1**) (50.0 g, 240 mmol, 1.00 equiv.) in 330 mL of dichloromethane, a solution of aluminum trichloride (56.0 g, 420 mmol, 1.75 equiv.) and acetyl chloride (30.0 mL, 33.0 g, 420 mmol, 1.75 equiv.) in 330 mL of dichloromethane was slowly added at −50 °C. The combined solutions were stirred for 3 h at −20 °C and then poured on ice. After stirring until discoloration, the phases were separated. The aqueous phase was acidified with hydrochloric acid (12 M in water) and extracted with 3 × 100 mL of dichloromethane. The combined organic phases were then washed with 3 × 100 mL of 1 M NaOH_aq_ and 250 mL of brine. Afterward, the organic phase was dried over sodium sulfate, the solvent evaporated, and the crude solid was subjected to column chromatography on silica using pentane/EtOAc/CH_2_Cl_2_, 20:1:1 as the eluent to obtain 4-acetyl [2.2]paracyclophane (40.3 g, 161 mmol, 67% yield) as a colorless solid.

m.p.: 110 °C

R_f_ = 0.45 (n-pentane/EtOAc, 9:1)

^1^H NMR (400 MHz, CDCl_3_ [7.26 ppm], ppm) δ = 6.93 (d, *J* = 1.9 Hz, 1H), 6.66 (dd, *J* = 7.9, 1.9 Hz, 1H), 6.58–6.45 (m, 4 H), 6.38 (dd, *J* = 7.9, 1.9 Hz, 1H), 4.05–3.90 (m, 1H), 3.26–3.10 (m, 4 H), 3.09–2.96 (m, 2 H), 2.90–2.78 (m, 1H), 2.47 (s, 3H).
^13^C{^1^H} NMR (101 MHz, CDCl_3_ [77.16 ppm], ppm) δ = 200.4 (C_q_), 141.7 (C_q_), 140.4 (C_q_), 139.9 (C_q_), 139.3 (C_q_), 138.0 (C_q_), 136.5 (+, CH), 136.5 (+, CH), 134.3 (+, CH), 133.2 (+, CH), 133.0 (+, CH), 132.2 (+, CH), 131.3 (+, CH), 36.2 (−, CH_2_), 35.3 (−, CH_2_), 35.3 (−, CH_2_), 35.0 (−, CH_2_), 28.9 (+, CH_3_).
MS (EI, 70 eV, 30 °C), *m*/*z* (%): 250 (89) [M]^+^, 146 (100), 145 (62), 131 (16), 105 (26), 104 (77), 103 (31), 78 (18), 77 (18).
HRMS–EI *(m*/*z)*: [M]^+^ calcd for C_18_H_18_O 250.1352; found 250.1351.
IR (ATR, ṽ) = 3029 (w), 3007 (w), 2985 (w), 2951 (w), 2921 (m), 2887 (w), 2849 (w), 2776 (w), 2766 (w), 1679 (vs), 1643 (w), 1589 (w), 1551 (w), 1494 (w), 1483 (w), 1446 (w), 1431 (w), 1409 (w), 1349 (s), 1320 (w), 1296 (w), 1265 (vs), 1239 (m), 1203 (m), 1184 (m), 1176 (s), 1162 (m), 1126 (w), 1092 (w), 1065 (w), 1018 (w), 983 (w), 952 (m), 941 (w), 918 (w), 901 (s), 853 (vs), 805 (m), 792 (s), 730 (s), 710 (s), 686 (w), 647 (s), 615 (vs), 599 (s), 562 (w), 534 (w), 510 (vs), 493 (m), 458 (w), 431 (w), 419 (w), 395 (m), 384 (w) cm^−1^.

Additional information on the chemical synthesis is available in the Chemotion repository at https://dx.doi.org/10.14272/reaction/SA-FUHFF-UHFFFADPSC-BMIQCMMQLW-UHFFFADPSC-NUHFF-NUHFF-NUHFF-ZZZ.1 (accessed on 16 May 2024).

Additional information on the analysis of the target compound is available in the Chemotion repository at https://dx.doi.org/10.14272/BMIQCMMQLWBYIU-UHFFFAOYSA-N.3 (accessed on 16 May 2024).

**(rac)/(S_p_)-1-(4-[2.2]Paracyclophanyl)ethanol (3)** [15]

Lithium aluminum hydride (7.58 g, 200 mmol, 5.00 equiv.) was dissolved in dry THF (250 mL). 4-Acetyl [2.2]paracyclophane (**2**) (10.0 g, 40.0 mmol, 1.00 equiv.) was added portion-wise. After 15 h at 50 °C, water was carefully added, and the mixture was diluted with ethyl acetate. The phases were separated, and the aqueous layer was extracted with ethyl acetate (3 × 100 mL). The combined organic layers were washed with sodium chloride (sat. aq. solution, 200 mL), dried over sodium sulfate, and the solvent was removed under reduced pressure. The crude solid was purified by flash column chromatography (silica, n-pentane/EtOAc, 5:1) to afford 1-(4-[2.2]paracyclophanyl)ethanol (6.82 g, 27.0 mmol, 68% yield) as a colorless solid.

${\left[\alpha \right]}_{D}^{20}$ (CHCl_3_, 11 mg·mL^−1^, deg·mL·dm^−1^·g^−1^) = +349.

m.p.: 85 °C

R_f_ = 0.34, 0.27 (n-pentane/EtOAc, 4:1)

^1^H NMR (400 MHz, CDCl_3_ [7.26 ppm], ppm) δ = 6.66–6.59 (m, 1H), 6.57–6.33 (m, 6 H), 4.94 (q, J = 6.4 Hz, 0.5H), 4.87 (q, J = 6.5 Hz, 0.5H), 3.66 (ddd, J = 13.0, 10.0, 2.5 Hz, 0.5H), 3.34 (ddd, J = 13.6, 10.0, 2.1 Hz, 0.5H), 3.22–2.97 (m, 6 H), 2.96–2.78 (m, 1H), 1.76 (br, s, 0.5H), 1.6 (d, J = 6.5 Hz, 1.5H), 1.42 (br, s, 0.5H), 1.3 (d, J = 6.4 Hz, 1.5H).
^13^C{^1^H} NMR (101 MHz, CDCl_3_ [77.16 ppm], ppm) δ = 144.9 (C_q_), 142.3 (C_q_), 140.5 (C_q_), 140.4 (C_q_), 139.8 (C_q_), 139.6 (C_q_), 139.6 (C_q_), 139.5 (C_q_), 138.7 (C_q_), 135.8 (+, CH), 135.2 (+, CH), 135.0 (C_q_), 133.8 (+, CH), 133.5 (+, CH), 133.2 (+, CH), 133.2 (+, CH), 132.7 (+, CH), 132.3 (+, CH), 132.1 (+, CH), 131.7 (+, CH), 130.1 (+, CH), 130.0 (+, CH), 129.8 (+, CH), 128.3 (+, CH), 68.1 (+, CH), 67.6 (+, CH), 35.4 (−, CH_2_), 35.4 (−, CH_2_), 35.4 (−, CH_2_), 35.3 (−, CH_2_), 34.9 (−, CH_2_), 34.5 (−, CH_2_), 33.5 (−, CH_2_), 33.3 (−, CH_2_), 25.9 (+, CH_3_), 21.3 (+, CH_3_).
MS (EI, 70 eV, 50 °C), *m*/*z* (%): 252 (62) [M]^+^, 237 (31), 130 (100), 129 (67), 105 (92), 104 (64).
HRMS–EI *(m*/*z)*: [M]^+^ calcd for C_18_H_20_O 252.1509; found 252.1510.
IR (ATR, ṽ) = 3332 (m), 3010 (w), 2983 (w), 2968 (w), 2945 (w), 2924 (m), 2890 (w), 2850 (w), 1592 (w), 1497 (w), 1485 (w), 1443 (w), 1412 (s), 1401 (m), 1367 (m), 1353 (w), 1290 (w), 1266 (w), 1224 (w), 1205 (w), 1183 (w), 1153 (w), 1146 (w), 1120 (w), 1077 (vs), 1060 (m), 1027 (vs), 956 (w), 935 (w), 901 (m), 887 (m), 850 (vs), 792 (m), 755 (w), 734 (w), 714 (vs), 681 (w), 653 (vs), 635 (s), 609 (vs), 565 (m), 526 (s), 504 (vs), 484 (vs), 460 (w), 435 (w), 422 (w), 399 (w), 384 (w) cm^−1^.

Additional information on the chemical synthesis is available in the Chemotion repository at https://dx.doi.org/10.14272/reaction/SA-FUHFF-UHFFFADPSC-IOOIGEUVTB-UHFFFADPSC-NUHFF-NUHFF-NUHFF-ZZZ (accessed on 16 May 2024).

Additional information on the analysis of the target compound is available in the Chemotion repository at https://dx.doi.org/10.14272/IOOIGEUVTBOKRL-UHFFFAOYSA-N.1 (accessed on 16 May 2024).

**(S_p_)-4-Vinyl [2.2]paracyclophane (4)** [15]

Neutral aluminum oxide (7.70 g, 75.5 mmol, 7.50 equiv), activated at 155 °C for 18 h under high vacuum, was suspended in toluene (100 mL) together with (*S*_p_)-1-(4-[2.2]paracyclophanyl)ethanol (**3**) (2.54 g, 10.1 mmol, 1.00 equiv). The suspension was refluxed at 120 °C for 3 days using a Dean–Stark trap. After evaporation of the solvent filtering over a silica plug using n-pentane/EtOAc, 4:1 as eluent yielded (*S*_p_)-4-vinyl [2.2]paracyclophane (2.31 g, 9.87 mmol, 98% yield) as a colorless solid.

${\left[\alpha \right]}_{D}^{20}$ (CHCl_3_, 9 mg mL^−1^, deg mL dm^−1^ g^−1^) = +318.

m.p.: 82 °C.

R_f_ = 0.86 (cyclohexane/ethyl acetate, 4:1).

^1^H NMR (400 MHz, CDCl_3_ [7.26 ppm], ppm) δ = 6.82 (dd, *J* = 17.4, 10.9 Hz, 1H), 6.73 (dd, *J* = 7.8, 1.8 Hz, 1H), 6.58–6.47 (m, 4 H), 6.47–6.39 (m, 2 H), 5.56 (dd, *J* = 17.4, 1.4 Hz, 1H), 5.30 (dd, *J* = 10.9, 1.4 Hz, 1H), 3.50 (ddd, *J* = 13.6, 9.9, 1.9 Hz, 1H), 3.19–2.91 (m, 6 H), 2.82 (ddd, *J* = 13.5, 10.3, 6.7 Hz, 1H).
^13^C{^1^H} NMR (101 MHz, CDCl_3_ [77.16 ppm], ppm) δ = 139.9 (C_q_), 139.5 (C_q_), 139.5 (C_q_), 138.1 (C_q_), 137.9 (+, CH), 135.3 (+, CH), 134.9 (+, CH, *C*_Ar_), 133.2 (+, CH, *C*_Ar_, 2C), 132.1 (+, CH), 131.9 (+, CH), 130.3 (+, CH), 129.7 (+, CH), 114.4 (−, CH2), 35.6 (−, CH_2)_, 35.3 (−, CH_2_), 34.8 (−, CH_2_), 33.8 (−, CH_2_).
MS (EI, 70 eV, 30 °C), *m*/*z* (%): 234 (33) [M]^+^, 130 (49), 129 (100), 115 (28), 104 (20).
HRMS–EI *(m*/*z)*: [M]^+^ calcd for C_18_H_18_ 234.1403; found 234.1404.
IR (ATR, ṽ) = 3030 (w), 3007 (m), 2948 (w), 2925 (s), 2918 (s), 2890 (m), 2847 (s), 1619 (w), 1589 (w), 1497 (w), 1482 (m), 1434 (m), 1417 (m), 1409 (m), 1203 (w), 1183 (w), 986 (s), 938 (w), 905 (vs), 880 (w), 864 (vs), 810 (m), 793 (s), 748 (vs), 715 (vs), 688 (w), 670 (vs), 628 (s), 569 (m), 555 (m), 507 (vs), 460 (w), 439 (w), 422 (w), 387 (w) cm^−1^.

Additional information on the chemical synthesis is available in the Chemotion repository at https://dx.doi.org/10.14272/reaction/SA-FUHFF-UHFFFADPSC-ITQUKLNMBH-UHFFFADPSC-NUHFF-NUHFF-NUHFF-ZZZ.1 (accessed on 16 May 2024).

Additional information on the analysis of the target compound is available in the Chemotion repository at https://dx.doi.org/10.14272/ITQUKLNMBHVYJB-UHFFFAOYSA-N.2 (accessed on 16 May 2024).

**(S_p_)-(4-[2.2]Paracyclophanyl)oxirane (5)** [16]

To a magnetically stirred solution of (*S*_p_)-4-vinyl [2.2]paracyclophane (**4**) (100 mg, 427 μmol, 1.00 equiv) in acetone (5 mL) at 21 °C, dimethyldioxirane (DMDO) in acetone (0.0798 M, 21.4 mL) was added slowly. After completion of the reaction, as indicated by thin-layer chromatography (around 30 min), the solvent was removed in vacuo. The yellow oil was dissolved in a small amount of DCM, and impurities were precipitated by adding pentane. Afterward, the solvent was removed under reduced pressure. Filtration through a short silica plug (n-pentane/EE 4:1) led to (*S*_p_)-(4-[2.2]Paracyclophanyl)oxirane (53.0 mg, 212 μmol, 50% yield).

${\left[\alpha \right]}_{D}^{20}$ (CHCl_3_, 13 mg mL^−1^, deg mL dm^−1^ g^−1^) = +124.

m.p.: 79 °C.

R_f_ = 0.33, 0.42 (n-pentane/ethyl acetate 10:1).

^1^H NMR (400 MHz, CDCl_3_ [7.26 ppm], ppm) δ = 6.81–6.75 (m, 0.5H), 6.62 (dd, J = 8.0, 1.8 Hz, 0.5H), 6.59–6.46 (m, 4 H), 6.44 (d, J = 6.4 Hz, 0.5H), 6.36 (dd, J = 7.8, 1.8 Hz, 0.5H), 6.28 (d, J = 1.9 Hz, 0.5H), 6.16 (d, J = 1.8 Hz, 0.5H), 3.93–3.83 (m, 1H), 3.53 (dddd, J = 25.0, 12.9, 10.0, 2.5 Hz, 1H), 3.24 (td, J = 4.9, 4.2, 3.1 Hz, 1H), 3.21–2.83 (m, 7.5H), 2.53 (dd, J = 5.7, 2.6 Hz, 0.5H).
^13^C{^1^H} NMR (101 MHz, CDCl_3_ [77.16 ppm], ppm) δ = ^13^C NMR (101 MHz, CDCl3 [77.16 ppm], ppm) δ = 140.7 (C_q_, 0.5 C), 140.3 (C_q_, 0.5 C), 139.7 (C_q_, 0.5 C), 139.6 (C_q_, 0.5 C), 139.5 (C_q_, 0.5 C), 139.1 (C_q_, 0.5 C), 139.0 (C_q_, 0.5 C), 138.3 (C_q_, 0.5 C), 136.9 (C_q_, 0.5 C), 135.8 (C_q_, 0.5 C), 135.2 (+, CH, 0.5 C), 134.8 (+, CH, 0.5 C), 133.4 (+, CH), 133.2 (+, CH, 0.5 C), 132.9 (+, CH, 0.5 C), 132.8 (+, CH, 0.5 C), 132.4 (+, CH, 0.5 C), 132.3 (+, CH, 0.5 C), 132.0 (+, CH, 0.5 C), 131.5 (+, CH, 0.5 C), 130.1 (+, CH, 0.5 C), 129.3 (+, CH, 0.5 C), 129.1 (+, CH, 0.5 C), 51.4 (+, CH), 50.7 (−, CH2, 0.5 C), 50.3 (−, CH_2_, 0.5 C), 35.5 (−, CH_2_, 0.5 C), 35.5 (−, CH_2_, 0.5 C), 35.4 (−, CH_2_, 0.5 C), 35.3 (−, CH_2_, 0.5 C), 35.3 (−, CH_2_, 0.5 C), 34.9 (−, CH_2_, 0.5 C), 33.4 (−, CH_2_, 0.5 C), 33.2 (−, CH_2_, 0.5 C).
MS (EI, 70 eV, 70 °C), *m*/*z* (%): 250 (18), 118 (92), 117 (27), 115 (23), 105 (19), 104 (100), 103 (24), 91 (18), 78 (18).
HRMS–EI *(m*/*z)*: [M]^+^ calcd for C_18_H_18_O 250.1352; found 250.1353.
IR (ATR, ṽ) = 3030 (w), 3007 (w), 2986 (w), 2922 (vs), 2893 (m), 2850 (s), 2704 (w), 1720 (vs), 1686 (w), 1672 (w), 1657 (w), 1592 (w), 1499 (m), 1492 (m), 1453 (w), 1435 (m), 1412 (m), 1385 (w), 1289 (w), 1261 (w), 1239 (w), 1217 (w), 1207 (w), 1183 (w), 1154 (w), 1142 (w), 1128 (w), 1101 (w), 1088 (w), 1078 (w), 1038 (w), 1007 (w), 984 (w), 960 (w), 939 (m), 928 (w), 905 (s), 882 (vs), 877 (vs), 858 (s), 847 (s), 796 (vs), 745 (m), 730 (s), 715 (vs), 681 (w), 647 (vs), 591 (m), 568 (m), 514 (vs), 499 (vs), 465 (m), 452 (m), 432 (w), 414 (w), 387 (w) cm^−1^.

Additional information on the chemical synthesis is available in the Chemotion repository at https://dx.doi.org/10.14272/reaction/SA-FUHFF-UHFFFADPSC-QHARUYCUDO-UHFFFADPSC-NUHFF-NUHFF-NUHFF-ZZZ (accessed on 16 May 2024).

Additional information on the analysis of the target compound is available in the Chemotion repository at https://dx.doi.org/10.14272/QHARUYCUDOUFLM-UHFFFAOYSA-N.1 (accessed on 16 May 2024).

**Dimethyldioxirane (DMDO)** [16]

Sodium bicarbonate (96 g, 1.14 mol, 0.71 eq.) was suspended in a mixture of water (80 mL) and acetone (120 mL, 1.62 mol, 1.00 eq.) and chilled in an ice/water bath. This mixture was stirred for 15 min. After 15 min, the stirring was stopped, and Oxone (100 g, 163 mmol, 0.10 eq.) was added in a single portion. The reaction mixture was loosely covered, and the suspension was stirred vigorously for 30 min while still being cooled in the ice bath. After 30 min, the stir bar was removed from the reaction flask and rinsed with a small portion of distilled water. The reaction mixture’s flask was then attached to a rotary evaporator with the bath at 22 °C. The receiving flask was chilled in a dry ice/isopropanol bath, and a vacuum of 200 mbar was applied. After 25 min, the bath temperature was raised to 40 °C over 10 min. When the bath reached 40 °C, the distillation was stopped immediately by releasing the vacuum and raising the flask from the heated water bath. The pale-yellow acetone solution of DMDO was dried over sodium sulfate. The sodium sulfate was removed by filtration and rinsed with 10 mL and again 3 mL of acetone, and afterward, the volume of the DMDO solution was measured volumetrically. The concentration of the DMDO solution was calculated via oxidation of thioanisole in ^1^H-NMR (as shown in chapter 6.1.3). Concentrations of the solution varied between 0.0788 and 0.0988 mol/L. The solution was stored over 4 Å molar sieves under argon at −20 °C.

**Homopolymerization for poly(4-[2.2]paracyclophanyl)oxirane) P1-P8** [17]

The polymerizations were performed at 60 °C under argon in a crimp vial equipped with a magnetic stirrer. The vial was flamed under vacuum before the addition of (4-[2.2]paracyclophanyl)oxirane (**15**) and tetraalkylammonium halide (0.02 mol%). Then, toluene and the trialkylaluminum (0.06 mol%) catalyst were added. After 3 days, a small amount of ethanol was added to stop the reaction, and MeOH was added to precipitate the polymer. The polymers were collected by centrifugation and dried under reduced pressure. Analytical data for **P8** are listed below.

${\left[\alpha \right]}_{D}^{20}$ (CHCl_3_, 9 mg mL^−1^, deg mL dm^−1^ g^−1^) = +46.

^1^H NMR (400 MHz, CDCl_3_ [7.26 ppm], ppm) δ = 7.00–5.96 (m), 3.58–2.38 (m), 2.45–1.98 (m).
IR (ATR, ṽ) = 3439 (w), 3432 (w), 3427 (w), 3398 (w), 3390 (w), 3383 (w), 3374 (w), 3369 (w), 3357 (w), 3352 (w), 2952 (w), 2918 (vs), 2871 (w), 2850 (m), 1720 (w), 1591 (s), 1458 (vs), 1453 (vs), 1411 (s), 1364 (m), 1323 (m), 1261 (m), 1179 (w), 1156 (w), 1084 (s), 1057 (s), 1024 (vs), 959 (m), 939 (m), 899 (m), 873 (m), 798 (vs), 756 (m), 717 (vs), 697 (vs), 636 (vs), 625 (vs), 589 (vs), 579 (vs), 555 (vs), 548 (vs), 535 (vs), 513 (vs), 459 (vs), 452 (vs), 441 (vs), 425 (vs), 418 (vs), 408 (vs), 398 (vs), 384 (vs) cm^−1^.

Additional information on the chemical synthesis is available in the Chemotion repository at https://dx.doi.org/10.14272/reaction/SA-FUHFF-UHFFFADPSC-PYIWRTYVVX-UHFFFADPSC-NUHFF-NUHFF-NUHFF-ZZZ (accessed on 16 May 2024).

Additional information on the analysis of the target compound is available in the Chemotion repository at https://dx.doi.org/10.14272/PYIWRTYVVXWWHU-UHFFFAOYSA-N.1 (accessed on 16 May 2024).

**Copolymerization for poly(4-[2.2]paracyclophanyl)oxirane-co-phtalic anhydrid) P9+P10** [18]

A vial equipped with a magnetic stirring bar was charged with PPNCl (3.90 mg, 6.79 µmol, 0.0250 equiv), (4-[2.2]paracyclophanyl)oxirane (**15**) (68.0 mg, 272 µmol, 1.00 equiv), phthalic anhydride (40.2 mg, 272 µmol, 1.00 equiv), and dry toluene (0.9 mL). The vial was then placed into a preheated metal block. After 1 day reaction time at 110 °C, the vial was removed from the block, and MeOH was added to precipitate the reaction mixture. The polymers were collected by centrifugation and dried under reduced pressure. Analytical data for **P10** are listed below.

${\left[\alpha \right]}_{D}^{20}$ (CHCl_3_, 12 mg mL^−1^, deg mL dm^−1^ g^−1^) = +18.

^1^H NMR (400 MHz, CDCl_3_ [7.26 ppm], ppm) δ = 8.15–7.34 (m), 6.94–6.07 (m), 3.69–3.41 (m), 3.41–2.70 (m).
IR (ATR, ṽ) = 3010 (w), 2924 (m), 2851 (w), 1718 (vs), 1595 (w), 1581 (w), 1489 (w), 1446 (w), 1411 (w), 1383 (vw), 1256 (vs), 1119 (vs), 1064 (vs), 1038 (s), 965 (w), 899 (w), 796 (m), 734 (s), 717 (s), 704 (m), 646 (w), 588 (w), 511 (m), 504 (m) cm^−1^.

Additional information on the chemical synthesis is available in the Chemotion repository at https://dx.doi.org/10.14272/reaction/SA-FUHFF-UHFFFADPSC-RQJMVWFPHP-UHFFFADPSC-NUHFF-NUHFF-NUHFF-ZZZ (accessed on 16 May 2024).

Additional information on the analysis of the target compound is available in the Chemotion repository at https://dx.doi.org/10.14272/RQJMVWFPHPJEEO-UHFFFAOYSA-N.1 (accessed on 16 May 2024).

### 2.3. Additional Information

Determination of the DMDO Concentration using ^1^H NMR Spectroscopy

**Figure 1:**
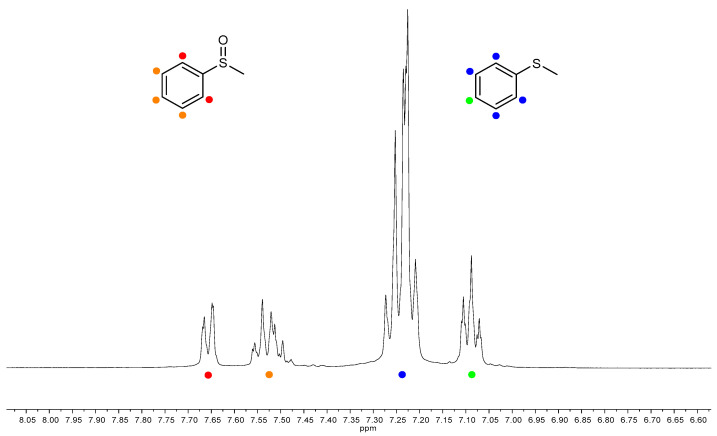
^1^H NMR spectrum (aromatic region) of a mixture of thioanisole and its sulfoxide.

The concentration was determined by non-stoichiometric oxidation of thioanisole according to the following procedure: A mixture of 0.1 mL thioanisole (106 mg, 0.853 mmol) and 1.2 mL acetone-d_6_ was cooled to 0 °C. Then, 3 mL of the obtained DMDO solution was added to the reaction mixture and stirred for 10 min. Afterward, 0.6 mL of this solution was transferred to an NMR tube. Analysis of the ^1^H NMR spectrum via signal integration of the sulfoxide phenyl protons at 7.4–7.7 ppm against the thioanisole phenyl protons at 7.0–7.3 ppm, following the example of Taber et al. [19] allowed for the determination of the ratio of oxidized product to the excess of thioanisole (Figure 1). The aromatic protons were chosen because they were more isolated than the methyl groups, which were not fully separated from the acetone and water signals.

The concentration of the *DMDO* solution was calculated according to Equation (1):

(1) $$c\left(DMDO\right)=\frac{n_{\mathrm{Thioanisole}}}{\frac{\sum I_{\mathrm{Thioanisole}}}{\sum I_{\mathrm{Sulfoxide}}}\times 3\;\mathrm{mL}}$$

*n*_Thioanisole_: the amount of substance in 0.1 mL thioanisole (0.853 mmol).

Σ*I*_Thioanisole_: sum of the integrals corresponding to the hydrogen signals of thioanisole, as shown in Figure 1 (blue and green signals).

Σ*I*_Sulfoxide_: sum of the integrals corresponding to the hydrogen signals of thioanisole, as shown in Figure 1 (red and orange signals).

## 3. Results

### 3.1. Synthesis and Stereochemical Analysis of the Planar Chiral PCP-derived Monomer

**Scheme 1:**
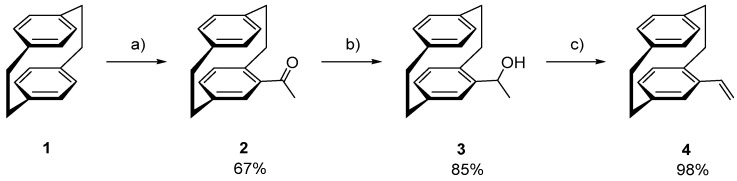
Stepwise synthesis of 4-vinyl [2.2]paracyclophane (**4**) via 4-acetyl [2.2]paracyclophane (**2**) and 1-(4-[2.2]paracyclophanyl)ethanol (**3**) according to our group [15]. Conditions: (**a**) acetyl chloride, AlCl_3_, DCM, −20 °C, 3 h; (**b**) LiAlH_4_, dry THF, 50 °C, 18 h; and (**c**) Al_2_O_3_, toluene, 120 °C, 18 h [15].

**Scheme 2:**
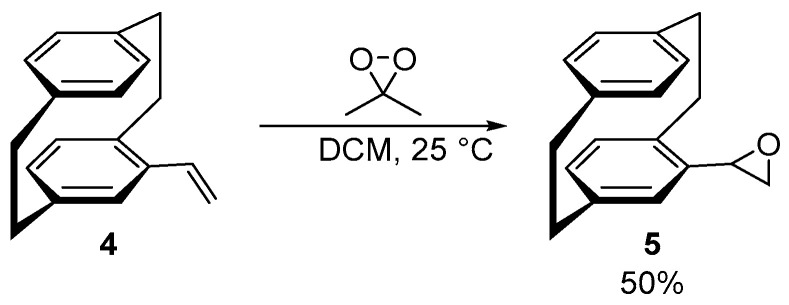
Synthesis of (4-[2.2]paracyclophanyl)oxirane (**5**) using DMDO as the oxidizing agent [16]. Conditions: DMDO, DCM, 25 °C, 30 min.

The racemic precursor **4** was synthesized in a three-step synthesis route starting from the unsubstituted PCP core with a total yield of 56% (Scheme 1). To receive an enantiomerically pure precursor, the planar chirality was enriched (ee > 95%) by a kinetic resolution previously published by our group [15].

(4-[2.2]Paracyclophanyl)oxirane (**5**) was synthesized via oxidation of **4** according to a modified procedure by Mikula et al. [16]. This modification included a washing step to remove nucleophilic aqueous compounds from the solution before evaporation of the solvent since the heat of the water bath combined with remaining nucleophiles was sufficient to decompose epoxide **5**. Other epoxidations, such as oxidation via *m*-chloroperbenzoic acid, could not be applied successfully because of the sensitivity of the epoxide **5**. The mild conditions of using DMDO as the oxidant led to the desired (4-[2.2]paracyclophanyl)oxirane (**5**) (Scheme 2). 

Because of its lability towards acids and nucleophiles, the purification of the epoxide had to be performed under mild conditions. Purifying and separating the diastereomers was impossible using a full-size column without decomposition. Washing and extraction, followed by a quick column (less than three minutes on the stationary phase), led to pure monomer **5,** maintaining yields up to 50%. The planar chirality was preserved over its synthetic pathway (124 $\frac{deg\cdot mL}{g\cdot dm}$ for **5**). Control over the epoxide’s central chirality was not achieved with standard methods and proved unnecessary for this work’s goal of synthesizing a chiral polymer. Therefore, the used chiral monomers were only enriched to >95% ee planar chirality, achieved by the aforementioned kinetic resolution.

### 3.2. Homopolymerization

**Scheme 3:**
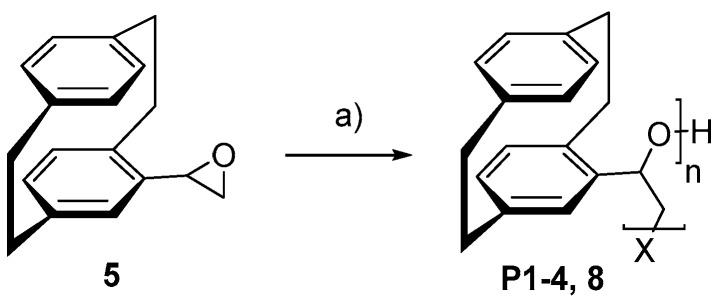
Polymerization of (4-[2.2]paracyclophanyl)oxirane (**5**) via an activated monomer strategy to yield poly(4-[2.2]paracyclophanyl)oxirane) (**P1-4 + P8**). Conditions: (**a**) AlR^1^_3_, NR^2^_4_X, toluene, 60 °C, 3 d; 0.02 mol% initiator, 0.06 mol% catalyst. R^1^ = Et, *^iso^*Bu; R^2^ = Bu, Oct; X = Cl, Br [17].

The activated monomer strategy was used to homopolymerize the synthesized epoxide since it supports the controlled ring-opening polymerization of sterically hindered epoxides. Therefore, the tested initiating systems consisted of tetraalkylammonium halides as initiators in combination with trialkylaluminum catalysts (Scheme 3) [17].

The different polymerization approaches, tested with racemic **5**, resulted in similar oligomeric batches of the polyether poly(4-[2.2]paracyclophanyl)oxirane) with low polydispersity, as summarized in Table 1. Approaches deviating from the activated monomer strategy did not result in polymers. 

The conditions of polymerization **P4** were additionally carried out with the chiral (*S_p_*)-(4-[2.2]paracyclophanyl)oxirane. The resulting polyether **P8** was isolated with an M_n_ of 1.90 kDa and a polydispersity of 1.19 in a yield of 13%. The chirality of **P8** was verified by measuring the specific rotation. During the polymerization, the specific rotation of the monomer decreased from 124 to 46 $\frac{\deg\cdot mL}{g\cdot dm}$. This possibly results from the overlaying non-defined polymeric architecture and the possibility of supramolecular chirality, and its control has to be targeted in future works.

### 3.3. Copolymerization

**Scheme 4:**
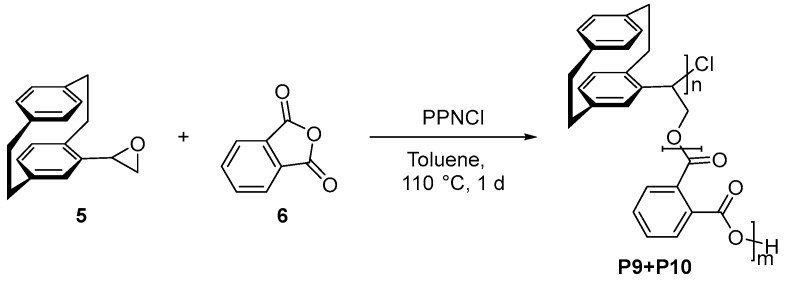
Statistical copolymerization of (4-[2.2]paracyclophanyl)oxirane (**5**) with phthalic anhydride (**6**) to yield the achiral and chiral form of the polyester poly(4-[2.2]paracyclophanyl) oxirane-co-phthalic anhydride (**P9 + P10**) [18]. Conditions: Bis(triphenylphosphine)iminium chloride (PPNCl), toluene, 110 °C, 1 d.

The epoxide monomer was also copolymerized to extend the possibilities of functionalization. Currently, many different comonomers for epoxides are known, such as CO_2_, [20] CS_2_, [21] isocyanates [22], and anhydrides [23]. The polymerization of epoxides with anhydrides leads to polyesters, representing a central class of polymers. A prominent representative is poly(ethylene terephthalate) (PET), used for various applications such as fibers, bottles, or other packaging applications [24]. For the copolymerization with epoxide **5**, phthalic anhydride (**6**) was used as the comonomer in a ratio of 1:1 since it was also compatible with the aromatic styrene oxide (Scheme 4) [18].

**Figure 2:**
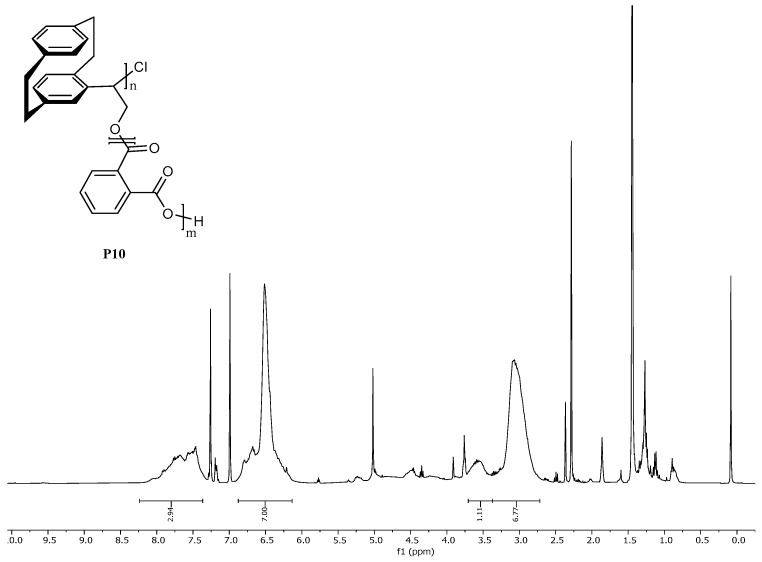
^1^H NMR of polyester **P10**.

In the ^1^H NMR spectrum of the obtained polyester poly(4-[2.2]paracyclophanyl) oxirane-co-phthalic anhydride, broad signals in the region of the expected shifts for both monomers are visible (Figure 2). Signals at 2.65–3.30 ppm and 3.40–3.70 ppm can be assigned to the CH_2_ bridge protons, while signals at 6.15–6.90 ppm correspond to the aromatic protons of the PCP core. The signals in the 7.29–8.20 ppm range can be assigned to the aromatic protons of the phthalic anhydride. The integral values suggest a 1:0.75 ratio (epoxide–anhydride). This polymerization was also performed with (*S*_p_)-(4-[2.2]paracyclophanyl)oxirane and the results of both are summarized in Table 2.

The resulting polymer **P10** showed a specific rotation of 18 $\frac{\deg\cdot mL}{g\cdot dm}$, which is a bit lower than half the value of the homopolymer **P8**. The decrease in optical activity aligns with the lower ratio of the chiral comonomer in the ^1^H NMR.

## 4. Summary

Within this work, we synthesized a chiral [2.2]paracyclophane-based epoxide monomer, which was then used for homo- and copolymerization with phthalic anhydride. It was shown that the chirality of the monomer was successfully transferred onto the resulting polymers. In addition to the homopolymerization, we demonstrated the feasibility of copolymerization using phthalic anhydride as a model compound. With this, we successfully took the first step into a broad field of applications for the (4-[2.2]paracyclophanyl)oxirane monomer.

In further works, the synthesis of a monomer with additional central chirality and other chiral [2.2]paracyclophane-based polymers such as poly-4-vinyl [2.2]paracyclophane will be investigated.

## Figures and Tables

**Table 1:** Polymerization of (4-[2.2]paracyclophanyl)oxirane (**5**) with different combinations of the initiator and catalyst. Reaction conditions included 0.02 mol% initiator and 0.06 mol% catalyst in toluene at 60 °C for 3 days.

Entry	Initiator	Catalyst	M_n_ [kDa]	D	Yield [%]
**P1**	NBu_4_Cl	Al*^i^*Bu_3_	1.85	1.12	18
**P2**	NOct_4_Br	Al*^i^*Bu_3_	1.78	1.08	19
**P3**	NBu_4_Cl	AlEt_3_	1.92	1.08	20
**P4**	NOct_4_Br	AlEt_3_	1.79	1.09	17
**P5**	NBu_4_Cl	Al*^i^*Bu_3_	-	-	-
**P6**	*sec*-BuLi	Al*^i^*Bu_3_	-	-	-
**P7**	NOct_4_Br	Co(II)-salen *	-	-	-
**P8** (chiral)	NOct_4_Br	Al*^i^*Bu_3_	1.90	1.19	13

* *N*,*N’*-bis(3,5-di-tert-butylsalicylidene)-1,2-cyclohexanediaminocobalt(II).

**Table 2:** Results of the copolymerization between (4-[2.2]paracyclophanyl)oxirane (**5**) and phthalic anhydride (**6**) [18]. **P9** used racemic **5** and **P10** used (*S*_p_)-(**5**).

Entry	M_n_ [kDa]	D	Yield [%]
**P9**	1.85	1.12	18
**P10** (chiral)	1.29	1.14	20

## Data Availability

The data presented in this study are openly available in the repository Chemotion (https://www.chemotion-repository.net/ accessed on 16 May 2024) at https://dx.doi.org/10.14272/collection/PAK_2023-11-15 accessed on 16 May 2024.
